# Health-related quality of life after critical care—the emperor’s new clothes

**DOI:** 10.1186/s13054-020-03012-3

**Published:** 2020-06-08

**Authors:** Folke Sjöberg, Lotti Orwelius, Sören Berg

**Affiliations:** 1 Department of Intensive Care, County Council of Östergötland, Linköping, Sweden; 2 Department of Clinical and Experimental Medicine, Linköping University, Linköping, Sweden; 3 Department of Burns, Hand and Plastic Surgery, County Council of Östergötland, Linköping, Sweden; 4 Department of Medical and Health Sciences, Linköping University, Linköping, Sweden; 5 Department of Cardiothoracic and Vascular Surgery, Linköping University, and County Council of Östergötland, Linköping, Sweden

## Background

Early in the 1970s, studies about critical care started to focus on outcome measures other than mortality, as mortality rates had decreased, and it now only was relevant to a smaller number of patients, i.e., those dying [1]. Ever since, health-related quality of life (HRQoL) has been examined extensively and most researchers have stressed that it is significantly affected by having undergone critical care treatment [2].

Assessing long-term outcomes for ICU patients, there is a distinct reduction in HRQoL (mainly physical), which usually has recovered 6 months later for a majority of the patient [3, 4], albeit there are subgroups with remaining issues [5, 6]. This is significantly less pronounced for the mental and social well-being in most patients which reaches long-term levels (about 20–30% lower than those in most corresponding control populations) as early as 2 months after leaving hospital [4, 7, 8]. However, it is important to acknowledge that the SF-36 instrument most commonly used for HRQoL assessment is less specific, as compared to other instruments to depict mental disorders [5]. Attempts to adjust for this have been done using more specific instruments in the severely injured burns patients and a similar outcome as seen for SF-36 mental dimensions has been claimed [7].

**Fig. 1:**
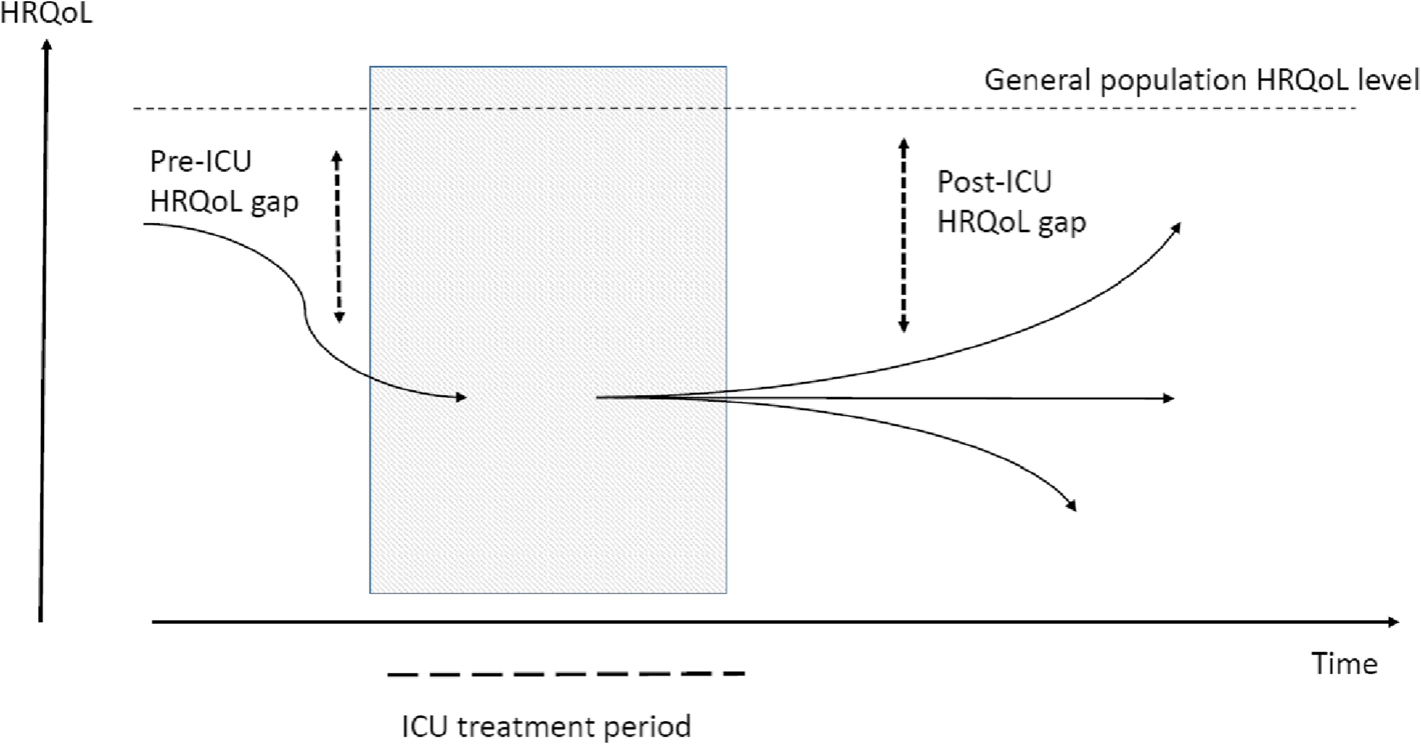
Figure illustrating the problem with lack of knowledge of the patients’ habitual HRQoL before the ICU period, the “pre-ICU gap,” when assessing the HRQoL after the ICU stay, the “post ICU gap.” Compared to the general population many, ICU patients have lower HQoL prior to ICU, due to both constitutional patient factors and factors related to the current disease requiring intensive care

Many studies indicate that comparing post-ICU patients with a regular, control population is not always adequate [7]. There are several reasons for this. The theory that a substantial reduction in HRQoL is caused by critical care treatment has been supported in the past by the exclusion of comorbidities and other pre-morbid factors. This misconception may be analogous to the tale of “The emperor’s new clothes.”^1^ (Fig. 1).

### Critical appraisal

So, what supports the supposition that ICU time only has a limited effect on the long-term HRQoL for the majority of ICU patients?

First, and most importantly, a recurring flaw in this evaluation is that ICU patients are being compared with healthy controls or a population lacking comorbidities.

Second, the time spent in ICU is bound to worsen pre-existing comorbidities or “frailty” recorded before ICU care [9].

Third, in ICU subgroups HRQoL, such seen for patients with COPD, no effect can be found when comparing it with that of patients with the same stage of COPD disease who have not been treated in ICU [10]. This is further supported by the finding that there was no difference in HRQoL when a comparison is made between patients treated in ICU compared to a disease-stratified hospitalized control population [11].

Fourth, a recent important finding is that a considerable portion of ICU patients (about 15%) is diagnosed with a chronic health condition while in ICU [12]. This leads to a combined number of comorbidities of former ICU patients, added to the 75% prevalence prior to the ICU period and, then reaching up to almost 90%, in total. It is also well shown that the pre ICU health trajectory can be extrapolated to the post ICU period [13].

Fifth, a significant portion of the studies does not include adjustments for age and sex [3]. Age adjustment is important, not least when comorbidities are not included because there is a clear collinearity between the two [14]. At times, age has even been used as a surrogate for comorbidities. A further problem with this is that in the oldest age group, comorbidities do not increase linearly but, rather, exponentially [14]. An interesting observation is that the HRQoL is good in a previous cohort of octogenarians, but it must then be stressed that this is most certainly a selected cohort, most often with less than “normal” comorbidity profile [6]. Regarding sex, there is a relative overrepresentation of single men in ICU. Single men have been shown as an independent risk factor for reduced HRQoL [7, 8].

Sixth, in studies adjusting for comorbidities, small effects on the outcome can be related to classic factors in intensive care (such as length of stay (LoS), SAPS3, APACHE IV, and time on a ventilator) [7]. Even in the case of PTSD, it was concluded that the most important risk factor for PTSD after a stay in ICU is the existence of a psychiatric diagnosis beforehand [12, 15].

Despite this, many studies of HRQoL after critical illness still lack analysis of comorbidities.

Finally, Skandinavian ICU LoS, as elsewhere, is relatively short for the majority of patients. One may wonder, can LoS as brief as a couple of days (median of less than 2) have an important impact on HRQoL that can last for 5 years? When longer LoS are included, which then constitutes a smaller portion of all ICU patients, little effect of LoS can be related to the reduced level of HRQoL [7]. The less-pronounced, negative impact of LoS is also seen in patients with burns. These patients, the majority of which is younger, stay considerably longer in ICU and have more instances of organ failure. Despite all of this, there are appreciable difficulties in showing an effect on their HRQoL as a result of the time spent in ICU when comorbidities are adjusted for.

The general ICU population has a plethora of specific diseases, each of which has its own particular characteristics and that will affect patients. A rational approach would be to assess long-term outcomes on this basis with person-centered measures that will discriminate between the disease-specific symptoms from the individual problems of the patients and which are looked for in risk (decreased HRQoL) prone subgroups.

## Conclusion

In conclusion, little of the final outcome in HRQoL for a majority of ICU patients can be connected to critical care itself. Instead, the most important effect on each individual is the result of their comorbidities or chronic conditions. Having said this, it needs to be stressed that there are subgroups that have ICU-related HRQoL effects that still deserve attention such as long-term ICU patients with, e.g., ARDS.

## Data Availability

Only referencing previous work.

## Footnotes

1 An idiomatic expression from the Danish fairy tale by Hans Christian Andersen that no one dares to tell the obvious truth if it is contradictory to what everyone else is saying is right, at the risk of being considered ignorant.
